# Dynamic proteomics reveals bimodal protein dynamics of cancer cells in response to HSP90 inhibitor

**DOI:** 10.1186/s12918-017-0410-8

**Published:** 2017-03-07

**Authors:** Anat Zimmer, Shlomit Amar-Farkash, Tamar Danon, Uri Alon

**Affiliations:** Department of Molecular Cell Biology, Weizmann Institute of Science, Rehovot, Israel

## Abstract

**Background:**

Drugs often kill some cancer cells while others survive. This stochastic outcome is seen even in clonal cells grown under the same conditions. Understanding the molecular reasons for this stochastic outcome is a current challenge, which requires studying the proteome at the single cell level over time. In a previous study we used dynamic proteomics to study the response of cancer cells to a DNA damaging drug, camptothecin. Several proteins showed bimodal dynamics: they rose in some cells and decreased in others, in a way that correlated with eventual cell fate: death or survival. Here we ask whether bimodality is a special case for camptothecin, or whether it occurs for other drugs as well. To address this, we tested a second drug with a different mechanism of action, an HSP90 inhibitor. We used dynamic proteomics to follow 100 proteins in space and time, endogenously tagged in their native chromosomal location in individual living human lung-cancer cells, following drug administration.

**Results:**

We find bimodal dynamics for a quarter of the proteins. In some cells these proteins strongly rise in level about 12 h after treatment, but in other cells their level drops or remains constant. The proteins which rise in surviving cells included anti-apoptotic factors such as DDX5, and cell cycle regulators such as RFC1. The proteins that rise in cells that eventually die include pro-apoptotic factors such as APAF1. The two drugs shared some aspects in their single-cell response, including 7 of the bimodal proteins and translocation of oxidative response proteins to the nucleus, but differed in other aspects, with HSP90i showing more bimodal proteins. Moreover, the cell cycle phase at drug administration impacted the probability to die from HSP90i but not camptothecin.

**Conclusions:**

Single-cell dynamic proteomics reveals sub-populations of cells within a clonal cell line with different protein dynamics in response to a drug. These different dynamics correlate with cell survival or death. Bimodal proteins which correlate with cell fate may be potential drug targets to enhance the effects of therapy.

## Background

Cancer drugs often kill some cells while other cells survive [1–5]. This stochastic outcome occurs even in clonal cells that are under identical conditions such as sister cells on the same plate. This stochastic resistance is non-genetic: The surviving cells, when re-plated, often give rise to populations that again show the same fraction of death versus survival in response to the drug [4, 6–8]. Inherited resistance evolves much slower, and usually occurs only after many such passages [3, 6, 9, 10]. The stochastic survival of cells may be one reason that cancer drugs do not always succeed in eliminating tumors, and understanding how some cells survive is therefore a pressing need.

In order to understand the molecular basis for the stochastic outcome of a drug, one needs to view the proteome in individual cells over time. Most existing proteomic methods average over millions of cells and therefore mask single-cell effects [1, 11]. Techniques for single-cell analysis based on immunostaining [12, 13] or transcriptomics [5] require fixing the cells and thus preclude studying the dynamics and eventual fate of each cell.

We have previously established a dynamic proteomics approach that addresses these issues and is able to follow proteins in single living human cancer cells over time. Dynamic proteomics is based on a library of cancer cell clones. Each clone expresses a full length tagged protein from its endogenous chromosomal locus [14–16]. We used this method to study the response of cells to the chemotherapy drug camptothecin (CPT) [2]. CPT is a topoisomerase poison which causes DNA damage [17] in dividing cells. Survival and death of different cells was found not to be due to cell-cycle differences. Instead, several proteins were found with different dynamics in individual cells, which correlated with cell fate. These proteins were called bimodal proteins: their level rose 20 h after CPT treatment in some cells, but decreased in other cells. Two proteins rose primarily in cells that survived, DDX5 and RFC1. Knocking down these proteins enhanced killing by CPT, suggesting a causal effect [2].

Here we ask whether bimodality of protein dynamics is specific to CPT, or whether it occurs also for another drug. For this purpose we used dynamic proteomics to analyze the response to a drug with a different mechanism of action, an HSP90 inhibitor (HSP90i). The HSP90i class of drugs inhibits the chaperone HSP90 and therefore interferes with protein degradation, which is thought to affect cancer cells more strongly than other cells [18–22].

We used dynamic proteomics to study how cells respond to an HSP90 inhibitor. We followed 100 proteins in space and time in living cancer cells following HSP90i treatment. We find 16 bimodal proteins whose protein dynamics are correlated with cell fate (survival/death). Seven of these were bimodal also in the previous CPT study. This indicates the formation of subpopulations of cells 12 h post treatments within a clonal cell line, long before death begins to at about 20 h. Unlike CPT, we find that HSP90i efficacy is correlated with the cell cycle. We further find localization changes of oxidative-stress response proteins which indicate that oxidative stress response is activated about 20 h after treatment. The dynamic proteomic approach thus suggests that bimodality may be a widespread response of cells to anti-cancer drugs, indicating that subpopulations with differential survival are induced by the drug many hours before death occurs.

## Methods

### Fluorescently-tagged protein clone library

The clones in this study were taken from the LARC library, in which proteins were fluorescently-tagged in their native locus and under their native regulation by CD-tagging as previously described [2, 23]. Briefly, H1299 (non-small cell lung carcinoma commercial cell line) cells were double tagged using the CD-tagging method, such that all clones contains a red fluorescent mCherry protein fused to a protein that is brighter in the nucleus and dimmer in the cytoplasm and is used for automated image analysis, and each clone expresses a yellow tag (eYFP or Venus) fused to the protein of interest. Each clone in the library originated from a single cell obtained by sorting for YFP fluorescence.

The H1299 cells that used to establish the LARC library originated from a commercial cell line, as described in Cohen et al. [2].

### Clone selection

For this study we chose a subset of clones from the LARC library. Clones were selected to provide coverage of different cellular pathways (such as: cell division, apoptosis, cell signaling), protein functions (such as: kinase, phosphatase, protease) and localization (such as: nuclei, nucleoli, cytoplasm, membrane). We chose 14 proteins which were found to be bimodal in the previous study of CPT (Table 1).

**Table 1:** list of Proteins used in this study, functional annotation based on Genecards [http://www.genecards.org/], and dynamic profile classification. Down–decrease in time, Down-up decrease in first 12 h followed by increase, up–increase over time

Protein name	Description	Dynamics
CKS2	CDC28 protein kinase, down regulated by p53, G1/S phase transition	Up
BAG1	blocks apoptosis, enhances anti-apoptotic effects of BCL2, Inhibits chaperone activity	
VPS26A	retromer complex, retrieve lysosomal enzyme receptors from endosomes to the Golgi	
H2AFV	H2A histone protein, nucleosome assembly	Down-up
UBE2K	ubiquitin enzyme E2, p53 degradation, NRF2 Oxidative Stress Response	
CACYBP	ubiquitin degradation, beta-catenin degradation, positive regulation of DNA replication	
RAB11A	RAS oncogene, membrane delivery during cytokinesis, protein transport, endocytosis	
TXN	Upregulate JUN & NFkB, apoptosis	
STMN1	destabilizing microtubules, mitotic spindle organization, activated by ERK	
STK24	serine/threonine kinase, apoptosis, upstream (MAPK) cascade	
RPS3A	ribosomal protein, translation, induction of apoptosis (BCL2 regulation)	
TXNRD1	response to oxidative stress, induces actin &tubulin polymerization, cell death	
FSCN1	actin-bundling protein, cell motility & migration, cell proliferation	
RBX1	E3 ubiquitin ligase, cell cycle progression, DNA repair	
SUMO1	ubiquitin-like, DNA repair, upregulate P53, nuclear transport, apoptosis, protein stability	
PAK2	p21 activated kinase, cytoskeleton reorganization, anti apoptosis	
TBCA	tubulin folding, stabilizing beta-tubulin	
SPCS1	proteolysis, metabolic process, removes signal peptides from proteins in the ER	
PRKAR2A	kinase, cAMP-dependent, lipid&glucose metabolism, signaling of GPCR, anti apoptosis	
CALM2	regulates the centrosome cycle and cytokinesis	
MAPK1	Proliferation, transformation- also known as ERK2, kinase	
IL3RA	interleukin 3 receptor, Immune response IL-3, Apoptosis, Jak-STAT signaling pathway	
PLEC1	intermediate filaments with MT & microfilaments, apoptosis, cell junction assembly	
PTPN11	Phosphatase, an upstream activator of Src & ras, Proliferation, transformation	
BAG2	BCL2-associated, Inhibits chaperones, apoptosis	
PCMTD2	protein modification, protein-L-isoaspartate(D-aspartate)O-methyltransferase domain	
VIL2	ezrin, intermediate between the PM and actin cytoskeleton, activate MET with CD44	
APAF1	initiates apoptosis	
MAP2K2	kinase, Activates the ERK1 and ERK2, Proliferation, transformation	
RFC1	replication & repair factor, subunit DNA polymerase, S phase of mitotic cell cycle	
TJP1	tight junction protein, apoptosis	
EEF2	elongation factor, protein synthesis	
DDX5	RNA helicases, splicing, cellular growth and division, P53 TF	
PSMB4	proteasome, cell cycle checkpoint	
MAPKAP1	mTORC2 Subunit, regulates cell growth & survival, regulate the actin cytoskeleton	
ENO1	Enolase, glycolysis, growth control, hypoxia tolerance, receptor, down regulate myc	
PBX3	Transcriptional activator that binds the sequence 5-ATCAATCAA-3	
RPL11	ribosomal protein L11, rRNA processing, translation	
LMNA	nuclear membrane fibrous, apoptosis, During mitosis disassembled, nuclear stability	
CDKN3	cell cycle arrest	
RPS3	ribosomal protein, translation, induction of apoptosis, repair UV-induced DNA damage	
FBL	snRNP, pre-r/tRNA processing, physical interaction with DDX5, coexpress with NCL	
AKAP8L	nuclear envelope breakdown & chromatin condensation. initiation of DNA replication	
PSMC4	proteasome cell cycle checkpoint	Down
HDAC2	histone deacetylase, cell growth arrest, differentiation and death	
PSMB7	proteasome cell cycle checkpoint	
PDCD5	induction of apoptosis	
RRAS	Regulates the organization of the actin cytoskeleton, RAS related, activates RAF	
DNMT1	CpG methylation, activated by JUN, STAT3, involved in P53 AKT paths	
K-alpha-1	tubulin, alpha 1b	
HMGA2	negative transcription regulation, chromatin organization, G1/S and G2/M transitions	
ARL3	cytokinesis and cilia signaling, microtubule binding, intracellular protein transport	
VCL	(F-actin)-binding protein, cell-matrix & cell-cell adhesion, cell morphology, locomotion	
CD44	MET activation, wound healing, inflammatory response, cell adhesion	
PSMA1	proteasome, cell cycle checkpoint, Immune response	
PGK1	phosphoglycerate kinase, glycolytic enzyme	
ILF2	immune response	
RPL22	ribosomal protein, component of the 60S subunit	
PPP1R2	carbohydrate metabolic process, phosphatase	
PKN1	JUN kinase activity, regulation of the actin cytoskeleton, TF	
HMGA1	regulation of transcription	
PTTG1	protein import into nucleus	
NPM1	chaperoning, histone assembly, cell proliferation, regulation of p53/TP53, ARF	
NASP	transporting histones into the nucleus, cell proliferation	
PFDN5	chaperone	
HAT1	internal protein A.A. acetylation, DNA packaging, HMGB1 signaling, DNA repair	
STK4	pro apoptosis regulator	
RTN4	pro apoptosis regulator	
PAWR	PRKC, pro apoptosis regulator	
SLBP	histone mRNA 3-end processing, mRNA export from nucleus	
CFLAR	CASP8 pro apoptosis gene	
YT521	RNA splicing (May be part of a signaling and alternative splicing)	
ANXA2	Calcium-regulated membrane-binding protein, signal transduction	
DNCH1	Microtubule molecular motors-cytoplasmatic.	

### Tissue Culture Media

Cells were grown in RPMI 1640 supplied with (+) L-Glutamine (GIBCO, catalog number 21875) medium supplemented with 10% Fetal Calf Serum (certified fetal bovine serum, membrane filtered, Biological Industries, catalog number 04-001-1A) and 0.05% Penicillin-Streptomycin antibiotics (Biological Industries, catalog number 03-031-1B), in incubators at 37 °C and 8% CO2.

### Drug addition

HSP90 inhibitor (MSC1972516 (EMD-614684)) [20], was dissolved in DMSO (hybri-max, D2650 Sigma) giving a stock solution of 1 mM. The drug was diluted to a final concentration of 1 μM by 1:1000 dilution in transparent growth medium (RPMI 1640, 0.05% Penicillin-Streptomycin antibiotics, 10% FCS, with L-Glutamine, lacking riboflavin and phenol red, Bet Haemek, Biological Industries Cat. No. 06-1100-26-1A). Cells were grown in 96-well plates (7 × 10^3^ cells per well) with normal (red) RPMI medium. Before each experiment, normal (red) RPMI medium was replaced by transparent RPMI 1640 (lacking riboflavin and phenol red to decrease medium fluorescence) with the desired drug (volume of 200ul per well). HGF (Hepatocyte Growth factor, Recombinant Human HGF, Catalog Number: 294-HGN, reconstituted at 50 μg/mL in sterile PBS containing 0.1% human or bovine serum albumin) was added at a final concentration of 100 ng/ml.

### Time-lapse microscopy

Time-lapse movies were obtained at 20× magnification, in IDEA Bio-Medical Ltd. WiScan system which includes Olympus inverted fluorescence microscope (modified IX71), live cell environmental incubators maintaining 37 °C (37-2 digital and Heating unit, PeCon, Germany, Leica #15531719), humidity and 8% CO_2_ (PeCon, GmbH, Germany #0506.000-230, Leica #11521733) and automated stage movement control (Corvus, ITK, GmbH, Germany); the stage was surrounded by a custom enclosure to maintain constant temperature, CO_2_ concentration, and humidity. Transmitted and fluorescent light paths were controlled by electronic shutters (Uniblitz, model VMM-D1, Rochester, NY); Fluorescent light sources were Short ARC Lamp HXP R 120w/45C VIS (OSRAM, Germany). iXon (Andor technology) cooled 14 bit CCD camera was used. The filters used were from Chroma Technology: Olympus Dual channel dichroic with excitation and emission filters mounted on fast filter wheels (Chroma set 490/20x, 577/25x, 535/30 m, 632/60 m).

Cells were visualized in 96-well optical glass-bottom plates (96 well optical CVG sterile w/lid Black, catalog number 164588 Thermo scientific nunc) coated with 10 μM fibronectin 0.1% (solution from bovine plasma, Sigma, Cat. No. F1141) diluted 1:100 in Dulbecco’s Phosphate Buffered Saline, PBS (Sigma, Cat. No. D8537). For each well, time-lapse movies were obtained at four fields of view. Each movie was taken for 2 days with 30 min resolution (100 time points). Each time point included three frames: a transmitted light image, and two fluorescent channels (red and yellow). In the present study we use the first 24 h of each movie.

### Image Analysis of Time-Lapse Movies

The image analysis software described in Cohen et al. [2] was used to analyze the time lapse movies in this study. The main steps in this software include background correction (flat field and background subtraction), segmentation, cell tracking, and automated identification of cell phenotypes (mitosis and cell death). Global image threshold (Otsu, 1979) followed by watershed segmentation was used to segment the nuclei and cytoplasm based on the red fluorescent images of the red tagged protein found in all clones. Tracking was performed by analysis of the movie from end to start and linking of each segmented cell to the cell in the previous image with the closest centroid, and other attributes like protein levels. Automated cell death identification algorithm utilized the morphological changes such as envelope breakdown, and cell roundness correlated with dying cells, and cells that undergo mitosis.

### Definition of clones with bimodal dynamics

For every clone, we calculated the slopes between every two time-points starting from t_0_ = 12 h after drug administration until t_1_ = 20 h after drug administration (total of 17 time points). We calculated for every cell the mean and median of the slopes between t_0_ and t_1_. Mean and median calculation showed similar results. Next we divided the cells in the clone into two groups: cells that had a positive slope on average–meaning that the protein level increased, and cells that had negative slope on average–meaning a decrease in protein level. We removed clones in which there were fewer than 10 cells in at least one of the two groups, or in which the movie quality was judged to be insufficient for analysis, remaining with 75 clones. We calculated the distribution of mean slopes in each group (positive and negative mean slope), and applied three tests for the null hypothesis H_0_ that these two groups are drawn from the same distribution: *t*-test, KS test and the nonparametric Mann Whitney test. To control for multiple hypothesis testing we applied the Benjaminy-Hochberg procedure with FDR = 0.1 to the results from all three tests. This result in a list of 25 proteins in which H_0_ can be rejected (*p* < 0.05 in all three tests).

### Bimodal dynamics and cell fate

We analyzed the 25 bimodal clones in terms of cell survival or death at 24 h. We divided the cells into two groups according to the fate of the cells, compared the slope distribution in these two groups, as described in the section above. We find that for 16 clones, cell fate significantly differed in the two groups (*t*-test, KS and MW tests, *p* < 0.05, Benjamini Hochberg corrected with FDR = 0.1).

## Results

**Fig. 1:**
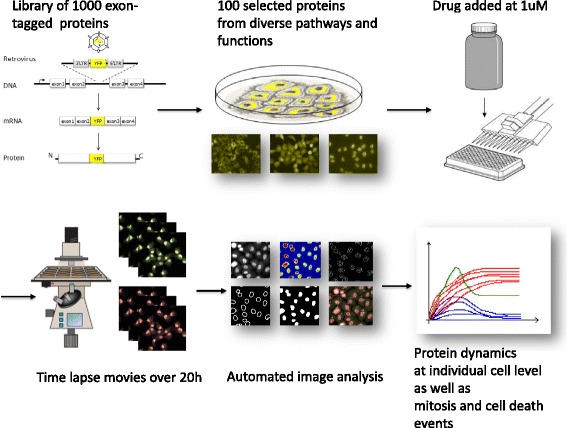
Workflow of dynamic proteomics in response to Hsp90 inhibitor drug: We used the LARC library of over 1000 H1299 cancer cell clones; in each clone a protein is tagged fluorescently at its natural locus using exon tagging. We selected 100 proteins in diverse pathways and functions, and assayed their dynamics in the presence of the drug, as well as in a control conditions. Time lapse movies over 20 h were conducted in 96-well format. Automated image analysis captured the protein dynamics at the individual cell level, as well as mitosis and death events for each cell

**Fig. 2:**
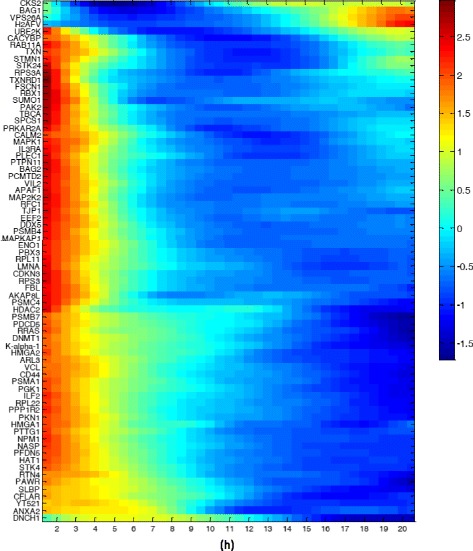
Dynamics of all proteins as a function of time after addition of the Hsp90 inhibitor. Protein dynamics was averaged over all cells, centered and normalized to mean zero and standard deviation one. *Red* denotes high relative levels and *blue*–low levels. Ordering of proteins is based on clustering the dynamics using Matlab

To study how cells respond to an HSP90 inhibitor we used dynamic proteomics. We measured the dynamics of 100 proteins involved in a wide range of cellular functions including signaling, growth and death, in response to 1 uM of an Hsp90 inhibitor (Merc Serrono MSC1972516) (proteins listed in Table 1). Cell death occurred 15 h+/−3 after drug addition. Protein dynamics were assayed using clones from the LARC library, described in [2]. In these clones of the H1299 lung-cancer cell line, proteins were tagged with YFP at their endogenous chromosomal location (Fig. 1) using a CD-tagging approach. Previous work showed that the clones express full length tagged proteins from their native locus with intact regulatory sequences. All clones also express red fluorescent proteins that aid fully-automated image analysis. Dynamics of protein level at a resolution of 30 min were examined for 24 h after drug addition in 96-well plates in a dedicated fluorescence microscopy system with controlled CO_2_ and temperature. Protein YFP level and localization, as well as cell mitosis and death events were automatically tracked and quantitated for hundreds of individual cells per clone as described [2] (Fig. 1). Protein YFP levels averaged over all cells showed diverse dynamics with most proteins dropping in levels on average and several other rising at late times (Fig. 2). Such dynamics that are averaged over all cells mask single cell-effects, to which we now turn.

### A quarter of the proteins show bimodal behavior, decaying in some cells and increasing in others, in a way that correlates with cell fate

**Fig. 3:**
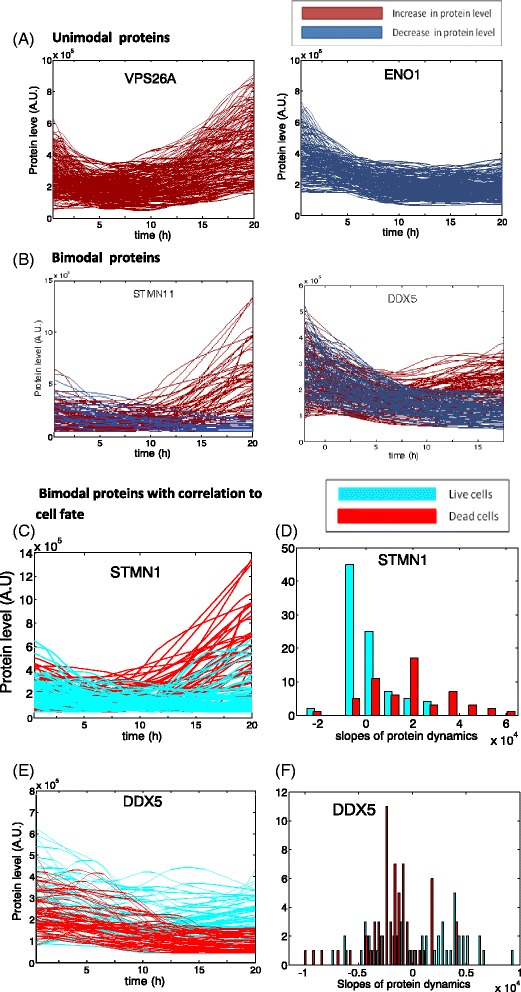
Several proteins show bimodal dynamics, increasing in some cells and decreasing in others, in a way that correlates strongly with cell survival or death. **a** Most proteins show dynamics which is unimodal–all cells follow the mean, with about two-fold variation around the mean. Shown are retromer VSP26, and the enzyme ENO1. **b** 25 proteins have bimodal dynamics. Some cells show a decrease in protein levels (*dark blue*); other cells show an increase after 12 h (*red*). Shown are the oncogene DDX5 and the mitotic spindle protein STMN1. **c** STMN1 dynamics correlate with cell survival or killing: cells in which STMN1 increases are preferentially killed. Surviving cells are in *light blue*, killed cells in *red*. **d** Histogram of slopes of STMN1 protein accumulation in the last 10 h (slope of linear regression of protein level as a function of time). Cells with a large slope (increase) preferentially are killed. **e** DDX5 dynamics correlate in an inverse way with survival or killing: cells in which DDX5 increases preferentially survive to the end of the movie. Surviving cells are in *light blue*, killed cells in *red*. **f** Histogram of slopes of DDX5 protein accumulation in the last 10 h (slope of linear regression of protein level as a function of time). Cells with a large slope (increase) preferentially survive

We studied the dynamics of the proteins at the individual cell level. We used the criteria of [2] to define bimodal dynamics: we computed the rate of accumulation of YFP protein level for each cell defined by the slope of the YFP curve over time. We separated the cells into two groups, with positive and negative slopes. Bimodal proteins were proteins from which the slope distribution of the two groups was significantly different as judged by using statistical tests (see Methods, *t*-test results shown in Table 2). We find that 25 out of 100 proteins (listed in Table 2) displayed a bi-modal response to HSP90i with expression increasing in some cells but decreasing in other cells 12 h after drug addition (Fig. 3).

**Table 2:** Bimodal Proteins, their functional annotation and their correlation to cell fate. Annotation is based on Genecards [http://www.genecards.org/]

Protein name	Description	Bimodal correlation to death	*P*-value
APAF1	initiates apoptosis	Increase correlates with death	3E-13
BAG1	blocks apoptosis, enhances anti-apoptotic effects of BCL2, Inhibits chaperone activity		8E-07
BAG2	BCL2-associated, Inhibits chaperones, apoptosis		2E-03
CACYBP	ubiquitin degradation, beta-catenin degradation, positive regulation of DNA replication		3E-08
CALM2	regulates the centrosome cycle and cytokinesis		3E-04
CKS2	CDC28 protein kinase, down regulated by p53, G1/S phase transition		2E-05
IL3RA	interleukin 3 receptor, Immune response IL-3, Apoptosis, Jak-STAT signaling pathway		5E-49
RAB11A	RAS oncogene, membrane delivery during cytokinesis, protein transport, endocytosis		2E-08
RBX1	E3 ubiquitin ligase, cell cycle progression, DNA repair		2E-02
STK24	serine/threonine kinase, apoptosis, upstream (MAPK) cascade		2E-02
STMN1	destabilizing microtubules, mitotic spindle organization, activated by ERK		7E-15
TXN	Upregulate JUN & NFkB, apoptosis		8E-27
DDX5	RNA helicases, splicing, cellular growth and division, P53 TF	Increase correlates with survival	1E-05
MAP2K2	kinase, Activates the ERK1 and ERK2, Proliferation, transformation		2E-05
RFC1	replication & repair factor, subunit DNA polymerase, S phase of mitotic cell cycle		9E-08
TBCA	tubulin folding, stabilizing beta-tubulin		2E-02
H2AFV	H2A histone protein, nucleosome assembly	no correlation with death	5E-04
LMNA	nuclear membrane fibrous, apoptosis, During mitosis disassembled, nuclear stability		5E-58
PAK2	p21 activated kinase, cytoskeleton reorganization, anti apoptosis		2E-10
PCMTD2	protein modification, protein-L-isoaspartate(D-aspartate)O-methyltransferase domain		7E-04
PLEC1	intermediate filaments with MT & microfilaments, apoptosis, cell junction assembly		1E-04
PRKAR2A	kinase, cAMP-dependent, lipid&glucose metabolism, signaling of GPCR, anti apoptosis		9E-05
PTPN11	Phosphatase, an upstream activator of Src & ras, Proliferation, transformation		1E-02
SPCS1	proteolysis, metabolic process, removes signal peptides from proteins in the ER		3E-19
TJP1	tight junction protein, apoptosis		2E-02

For 16 out of the 25 bimodal proteins the protein dynamics correlated with cell fate (death or survival at the end of the experiment). These proteins and their function are listed in Table 2. For 12 proteins, such as STMN1, a protein important for mitosis spindle organization, cells that increase the protein level are preferentially killed (*p* = 7e^−15^). Many of these proteins are not naturally associated with cell death, including proteins involved in mitosis and in anti-apoptotic functions. The increase of 4 proteins correlated with cell survival, including DDX5 and RCF1. For 9 of the bimodal proteins we found no significant correlation with cell death or cell survival.

Six of the bimodal proteins, DDX5, RFC1, BAG1, BAG2, SPCS1, CALM2 and PCMTD2 were previously identified as bimodal also for CPT. The other 18 bimodal proteins found here were not bimodal in CPT, suggesting that the stochastic mechanisms in the response to the two drugs have only partial overlap.

### Cells that attempt mitosis 12 h or more after drug addition are preferentially killed

**Fig. 4:**
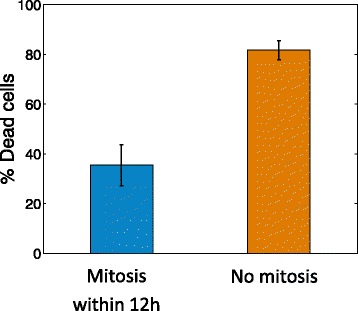
Cells which undergo mitosis 12 h after drug treatment are preferentially killed. Bar plot shows percentage of cells which show morphological correlate of cell death in the 20 h movie. Cells are binned into those that show mitosis in the first 12 h (*n* = 161 cells), and those that do not (*n* = 120 cells). Cells are from multiple movies with different clones. *Error bars* are standard errors

We next asked whether the cell-cycle phase at the moment of drug administration affects cell survival. We automatically detected mitosis events as described [2], using morphological changes of cells, primarily cell rounding (see Methods) [2, 14, 16, 24]. We find that cells that attempt mitosis 12 h or more after drug administration were not able to complete cell division and were preferentially killed (82%), whereas cells that show the morphological correlate of mitosis in the first 12 h were able to complete mitosis and were less frequently killed (35%), Fig. 4. This finding suggests that HSP90i effect depends on the cell-cycle stage of the cells. In contrast CPT showed no dependence on cell cycle stage, highlighting another difference between the drugs at the level of individual cell responses.

### Oxidative stress proteins increase in levels and enter the nucleus after drug addition

**Fig. 5:**
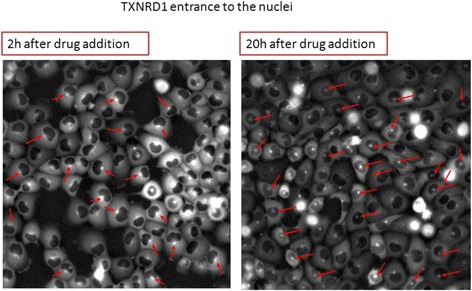
In response to the Hsp90i, the oxidative stress protein thioredoxin reductase TXNRD1 shows nuclear entry at late times. At early times (2 h) most cells show TXNRD1 localized to the ER, a bright dot near the nucleus (*red arrow*). At late times, most cells show a dot inside the nucleus (*red arrows*)

The dynamic proteomic approach also allows detection of localization changes in response to drugs. We observe translocations of thioredoxin (TXN) and thioredoxin reductase (TrxR), which show nuclear entry after 20 h (Fig. 5). This translocation occurs upon oxidative stress response [25–27]. A similar translocation of these two proteins occurred also for CPT [2]. Oxidative stress response is an off-target effect for these two drugs.

## Discussion

We asked whether bimodal protein dynamics, which were found for CPT, also occur for another drug with a different mechanism of action. We therefore used the dynamic proteomics approach to study how individual cancer cells respond to an HSP90 inhibitor. We find that a quarter of the proteins show bimodal dynamics: they rise in some cells and drop in others. This bimodality begins about 12 h post-drug, and indicates the existence of cell subpopulations. Most of the bimodal proteins have dynamics that correlate with eventual cell fate: death or survival.

The existence of cell subpopulations with different dynamics may explain why some cells survive and others die in response to the drug. The proteins that rise in correlation with cell survival may be potential drug targets to enhance HSP90i effects. In previous work two of the bimodal protein that rose preferentially in surviving cells, DDX5 and RFC1, were shown to increase effectiveness of CPT upon knockdown [2]. Two additional proteins were found in the present study that rose in correlation with survival: a MAPK that lies upstream of ERK1,2 which signals for proliferation, and a tubulin stabilizing protein TBCA. Future work can test the causal link of these proteins to survival under HSP90i.

Proteins that rose in cells that eventually die include mitosis and anti-apoptotic proteins. This result is unexpected because one might expect death-related proteins to rise in cells that are eventually killed rather than proteins associated with cell division and survival. One way to understand this finding is our observation that the cells that attempt mitosis more than 12 h after drug addition are preferentially killed. These cells may attempt to progress through the cell cycle and to complete mitosis in the face of the drug but fail to do so.

The present single cell approach also allowed us to identify localization changes indicative of oxidative stress. Thioredoxin and thioredoxin reductase both move to the nucleus 20 h post drug administration [24]. Such off-target effects may be relevant in understanding and addressing the toxicity of HSP90i drug candidates. Similar localization changes were observed also after camptothecin treatment [2].

Differences in cell cycle phase play a major role in cell-cell variability. We find that cells that attempted mitosis in the first 12 h of drug treatment were preferentially killed. This is in contrast with camptothecin that showed no measurable cell-cycle dependence. An advantage of the present approach is that it can directly view mitosis events. This non-invasive measurement avoids some of the concerns associated with chemical synchronization of cells to study cell-cycle effects. This advantage allowed dynamic proteomics to accurately detect cell-cycle dependent proteins in previous studies [28–30].

Future work can address the origin of the subpopulations found here. One possibility is that the subpopulations are formed dynamically after the drug is added. Another possibility is that subpopulations exist before the drug treatment due to genetic mutation or a slowly reversible epigenetic change [6]. Previous work on the H1299 cell line, the parental cell line for the present clones, suggests that it may harbor preexisting subpopulations. For H1299, cisplatin treatment of unsorted or stem cell marker CD44+ cells resulted in no significant increase of apoptotic cells compared to the untreated control, while CD44- cells showed significant increase in apoptosis after cisplatin treatment [31]. H1299 telomere-lengthening correlates with telomerase activity, but exhibits clonal variability in telomere-lengthening and telomerase activity [32]. Suboptimal doses of cisplatin on H1299 cells and re-plating select for an enhanced malignant phenotype [33]. These findings are in line with the idea that H1299 includes at least two different subclones with different drug-sensitivities.

It would be intriguing to further study the nature of the cell subpopulations in more detail. One can ask whether bimodal proteins go up and down in a coordinated way in each cell, or whether they are uncoordinated. This may require multi-color tagging of these proteins [28]. It would be important to test other drugs and cell lines in order to explore the generality of bimodality that correlates with cell fate.

## Conclusion

This study used single-cell dynamic proteomics to reveal sub-populations of cells that have different protein dynamics in response to a drug within a clonal cell line, in a way that correlates with cell survival or death. Bimodal proteins which correlate with cell fate may be potential drug targets to enhance the effects of therapy.

## Abbreviations

APAF1: Apoptotic Peptidase Activating Factor 1 (gene)
BAG1: BCL2 Associated Athanogene 1 (gene)
BAG2: BCL2 Associated Athanogene 2 (gene)
CALM2: Calmodulin 2 (gene)
CD-tagging: Central Dogma tagging (a method to label genes by integrating a DNA sequence coding for a fluorescent tag into the genome)
CPT: Camptothecin (an anti-cancer drug)
DDX5: DEAD (Asp-Glu-Ala-Asp)-Box Helicase 5 (gene)
ERK1,2: Extracellular signal-regulated kinases 1 or 2 (genes)
H1299: Human non-small cell lung carcinoma cell line
HSP90: Heat shock protein 90
HSP90i: Heat shock protein 90 inhibitor
LARC library: Library of annotated reporter cell clones, a collection of proteins which were fluorescently tagged in their endogenous chromosomal locus
MAPK: Mitogen-activated protein kinas (a type of protein kinase)
PCMTD2: Protein-L-Isoaspartate (D-Aspartate) O-Methyltransferase Domain Containing 2 (gene)
RFC1: Replication Factor C Subunit 1 (gene)
SPCS1: Signal Peptidase Complex Subunit 1 (gene)
STMN1: Stathmin 1 (gene)
TBCA: Tubulin Folding Cofactor A (gene)
TrxR: Thioredoxin reductase (gene)

## References

[1] Altschuler SJ, Wu LF (2010). Cellular Heterogeneity: Do Differences Make a Difference?. Cell.

[2] Cohen AA, Geva-Zatorsky N, Eden E, Frenkel-Morgenstern M, Issaeva I, Sigal A (2008). Dynamic proteomics of individual cancer cells in response to a drug. Science.

[3] Marusyk A, Almendro V, Polyak K (2012). Intra-tumour heterogeneity: a looking glass for cancer?. Nat Rev Cancer.

[4] Sharma SV, Lee DY, Li B, Quinlan MP, Takahashi F, Maheswaran S (2010). A chromatin-mediated reversible drug-tolerant state in cancer cell subpopulations. Cell.

[5] Tirosh I, Izar B, Prakadan SM, Wadsworth MH, Treacy D, Trombetta JJ (2016). Dissecting the multicellular ecosystem of metastatic melanoma by single-cell RNA-seq. Science.

[6] Flusberg DA, Sorger PK (2015). Surviving apoptosis: life–death signaling in single cells. Trends Cell Biol.

[7] Snijder B, Pelkmans L (2011). Origins of regulated cell-to-cell variability. Nat Rev Mol Cell Biol.

[8] Spencer SL, Gaudet S, Albeck JG, Burke JM, Sorger PK (2009). Non-genetic origins of cell-to-cell variability in TRAIL-induced apoptosis. Nature.

[9] Brock A, Chang H, Huang S (2009). Non-genetic heterogeneity—a mutation-independent driving force for the somatic evolution of tumours. Nat Rev Genet.

[10] Meacham CE, Morrison SJ (2013). Tumour heterogeneity and cancer cell plasticity. Nature.

[11] Wang D, Bodovitz S (2010). Single cell analysis: the new frontier in “omics.”. Trends Biotechnol.

[12] Yuan X, Curtin J, Xiong Y, Liu G, Waschsmann-Hogiu S, Farkas DL (2004). Isolation of cancer stem cells from adult glioblastoma multiforme. Oncogene.

[13] Zhang S, Balch C, Chan MW, Lai H-C, Matei D, Schilder JM (2008). Identification and Characterization of Ovarian Cancer-Initiating Cells from Primary Human Tumors. Cancer Res.

[14] Cohen AA, Kalisky T, Mayo A, Geva-Zatorsky N, Danon T, Issaeva I (2009). Protein Dynamics in Individual Human Cells: Experiment and Theory. PLoS ONE.

[15] Frenkel-Morgenstern M, Cohen AA, Geva-Zatorsky N, Eden E, Prilusky J, Issaeva I (2010). Dynamic Proteomics: a database for dynamics and localizations of endogenous fluorescently-tagged proteins in living human cells. Nucleic Acids Res.

[16] Issaeva I, Cohen AA, Eden E, Cohen-Saidon C, Danon T, Cohen L, et al. Generation of Double-Labeled Reporter Cell Lines for Studying Co-Dynamics of Endogenous Proteins in Individual Human Cells. PLoS ONE [Internet]. 2010;5. Available from: http://www.ncbi.nlm.nih.gov/pmc/articles/PMC2958823/. cited 25 Feb 2016. 10.1371/journal.pone.0013524 PMC2958823 20975952

[17] Hsiang YH, Hertzberg R, Hecht S, Liu LF (1985). Camptothecin induces protein-linked DNA breaks via mammalian DNA topoisomerase I. J Biol Chem.

[18] Sidera K, Patsavoudi E (2014). HSP90 Inhibitors: Current Development and Potential in Cancer Therapy. Recent Pat Anticancer Drug Discov.

[19] Proia DA, Bates RC (2014). Ganetespib and HSP90: translating preclinical hypotheses into clinical promise. Cancer Res.

[20] Porter JR, Fritz CC, Depew KM (2010). Discovery and development of Hsp90 inhibitors: a promising pathway for cancer therapy. Curr Opin Chem Biol.

[21] Jhaveri K, Modi S (2015). Ganetespib: research and clinical development. OncoTargets Ther.

[22] Whitesell L, Lindquist SL (2005). HSP90 and the chaperoning of cancer. Nat Rev Cancer.

[23] Sigal A, Danon T, Cohen A, Milo R, Geva-Zatorsky N, Lustig G (2007). Generation of a fluorescently labeled endogenous protein library in living human cells. Nat Protoc.

[24] Issaeva I, Cohen AA, Eden E, Cohen-Saidon C, Danon T, Cohen L (2010). Generation of Double-Labeled Reporter Cell Lines for Studying Co-Dynamics of Endogenous Proteins in Individual Human Cells. PLoS ONE.

[25] Lee K-H, Jang A-H, Yoo C-G (2015). 17-Allylamino-17-Demethoxygeldanamycin and the Enhancement of PS-341–Induced Lung Cancer Cell Death by Blocking the NF-κB and PI3K/Akt Pathways. Am J Respir Cell Mol Biol.

[26] Byrne BM, Welsh J (2005). Altered thioredoxin subcellular localization and redox status in MCF-7 cells following 1,25-dihydroxyvitamin D3 treatment. J Steroid Biochem Mol Biol.

[27] Karimpour S, Lou J, Lin LL, Rene LM, Lagunas L, Ma X (2002). Thioredoxin reductase regulates AP-1 activity as well as thioredoxin nuclear localization via active cysteines in response to ionizing radiation. Oncogene.

[28] Geva-Zatorsky N, Dekel E, Cohen AA, Danon T, Cohen L, Alon U (2010). Protein Dynamics in Drug Combinations: a Linear Superposition of Individual-Drug Responses. Cell.

[29] Farkash-Amar S, Eden E, Cohen A, Geva-Zatorsky N, Cohen L, Milo R (2012). Dynamic proteomics of human protein level and localization across the cell cycle. PLoS One.

[30] Sigal A, Milo R, Cohen A, Geva-Zatorsky N, Klein Y, Alaluf I (2006). Dynamic proteomics in individual human cells uncovers widespread cell-cycle dependence of nuclear proteins. Nat Methods.

[31] Leung EL-H, Fiscus RR, Tung JW, Tin VP-C, Cheng LC, Sihoe AD-L (2010). Non-Small Cell Lung Cancer Cells Expressing CD44 Are Enriched for Stem Cell-Like Properties. PLoS One.

[32] Savre-Train I, Gollahon LS, Holt SE (2000). Clonal heterogeneity in telomerase activity and telomere length in tumor-derived cell lines. Proc Soc Exp Biol Med Soc Exp Biol Med N Y N.

[33] Hsieh J-L, Lu C-S, Huang C-L, Shieh G-S, Su B-H, Su Y-C (2012). Acquisition of an enhanced aggressive phenotype in human lung cancer cells selected by suboptimal doses of cisplatin following cell deattachment and reattachment. Cancer Lett.

